# Polystyrene Micro- and Nanoplastic Exposure Triggers an Activation and Stress Response in Human Astrocytes

**DOI:** 10.3390/ijms262311273

**Published:** 2025-11-21

**Authors:** Sonia Kiran, Uvindu Thilanka, Yu Xue, Qing-Xiang Amy Sang

**Affiliations:** 1 Department of Chemistry and Biochemistry, Florida State University, Tallahassee, FL 32306, USA; skiran@fsu.edu (S.K.); ud24@fsu.edu (U.T.); yx21@fsu.edu (Y.X.); 2 Institute of Molecular Biophysics, Florida State University, Tallahassee, FL 32306, USA

**Keywords:** microplastics, nanoplastics, astrocytes, oxidative stress, inflammation, metabolism, cytotoxicity

## Abstract

Recent evidence indicates the presence of micro- and nanoplastics in the human brain, with higher accumulation observed in patients with dementia. However, their mechanistic effects on the human brain at the cellular level remain underexplored. Astrocytes play a crucial role in repairing neurons following injury. The dysfunction of these cells can lead to chronic inflammation, a hallmark of neurodegenerative diseases. Here, we investigated the cytotoxic responses of primary human astrocytes exposed to polystyrene particles of two representative sizes, 25 nm and 1 µm, at concentrations of 1 µg/mL and 5 µg/mL for 48 h. Flow cytometry and confocal microscopy revealed the accumulation of particles of both sizes within the cytoplasm. Functional assays revealed reduced cell viability and elevated lactate dehydrogenase release, indicating cytotoxic effects following microplastic exposure. Gene expression analysis showed significant upregulation of MAPK14 and SOD2, indicating oxidative stress activation, and increased expression of pro-inflammatory mediators IL-6, TNF-α, and NF-κB1. In parallel, GLUT1 transcripts and GLUT1-positive cell populations were markedly reduced, suggesting impaired glucose metabolism. Collectively, these findings demonstrate that microplastics disrupt astrocytic homeostasis by inducing oxidative, inflammatory, and metabolic disturbances, leading to a reactive yet metabolically compromised phenotype. This study demonstrates the cellular damage caused by microplastics in astrocytes, which may contribute to a cellular mechanism linking environmental pollutant exposure to adverse effects on human health.

## 1. Introduction

The ever-increasing utilization of plastic has resulted in plastic pollution as a critical global environmental and health concern. Discarded plastics in the environment undergo gradual fragmentation due to exposure to UV radiation, mechanical stress, and weathering processes, resulting in the formation of secondary microplastics, which are defined as particles measuring less than 5 mm in diameter and can further degrade into nanoparticle sizes that are smaller than 1 µm [1,2]. Among these, polystyrene (PS) is one of the most prevalent polymer types, commonly detected in food, drinking water, and indoor dust [3,4,5,6]. Human exposure occurs through ingestion, inhalation, and, to a lesser extent, dermal contact [7,8]. Recent in vivo studies have demonstrated that polystyrene micro- and nanoplastics (PS-MNPs) can enter various mammalian tissues and deposit in organs such as the lungs, liver, and brain [9,10,11,12].

Accumulating evidence indicates that PS-MNPs perturb cellular homeostasis by inducing oxidative stress, inflammation, and cytotoxicity, which may culminate in apoptosis or other forms of cell death [13,14,15,16,17,18]. In vivo experiments have demonstrated that PS-MNPs can penetrate the blood–brain barrier, leading to neuroinflammation, microglial activation, disrupted synaptic function, and even impaired learning and memory [12,19,20,21]. Autopsy and animal studies suggest that these particles accumulate preferentially in the brain, especially in individuals with dementia, which potentially contributes to neurodegenerative processes through mitochondrial dysfunction and disruption of proteostasis [21,22].

Astrocytes, as the fundamental glial cells in the central nervous system, play crucial roles in maintaining neural homeostasis, modulating immune responses, and preserving blood–brain barrier integrity [23]. Their metabolic versatility also makes them highly sensitive to environmental insults. Exposure to synthetic compounds, including MNPs, may dysregulate astrocytic metabolism and promote reactive astrogliosis, a condition that, while initially protective, can ultimately lead to glial scar formation [24] and impair neural recovery [24,25,26,27]. Recent studies using rodent or stem-cell-derived astrocyte models have shown that PS-MNP exposure alters gene expression related to oxidative stress and inflammatory signaling, elevates glial fibrillary acidic protein (GFAP) expression, and modulates the secretion of cytokines such as tumor necrosis factor α (TNF-α) and Interleukin-6 (IL-6) [27,28,29,30]. However, findings across models remain inconsistent, and there is limited understanding of how these mechanisms operate in normal human astrocytes.

To date, most investigations have relied on murine or stem-cell-derived astrocytes [12,27,28,29,31,32], while studies using primary normal human astrocytes (NHAs), the most physiologically relevant in vitro model, are lacking. NHAs retain native cellular functions and stress-response pathways, offering a more accurate representation of human brain responses. The present study examines how PS-MNPs of different sizes (25 nm and 1 µm) and concentrations (1 µg/mL and 5 µg/mL) influence cellular uptake, viability, and inflammatory and oxidative-stress responses in NHAs. These concentrations were selected to approximate exposure levels reported in human biological samples (~1.6 µg/mL) [33] (Supplementary Table S1). By integrating cytotoxicity assays with analyses of mRNA and protein markers related to oxidative stress (SOD2: Superoxide Dismutase 2, MAPK14: Mitogen-Activated Protein Kinase 14), astrocyte activation (GFAP: Glial Fibrillary Acidic Protein, STAT3: Signal Transducer and Activator of Transcription 3), neuroprotection (BDNF: Brain-Derived Neurotrophic Factor, GLUT1: Glucose Transporter 1), and inflammation (IL-6: Interleukin-6, TNF-α: Tumor Necrosis Factor Alpha, NF-κB1: Nuclear Factor Kappa B Subunit 1, TREM2: Triggering Receptor Expressed on Myeloid cells 2), this study aims to clarify how PS-MNPs modulate astrocyte function and contribute to potential neurotoxic outcomes..

## 2. Results

### 2.1. Characterization of PS-MNPs Using Dynamic Light Scattering

**Figure 1:**
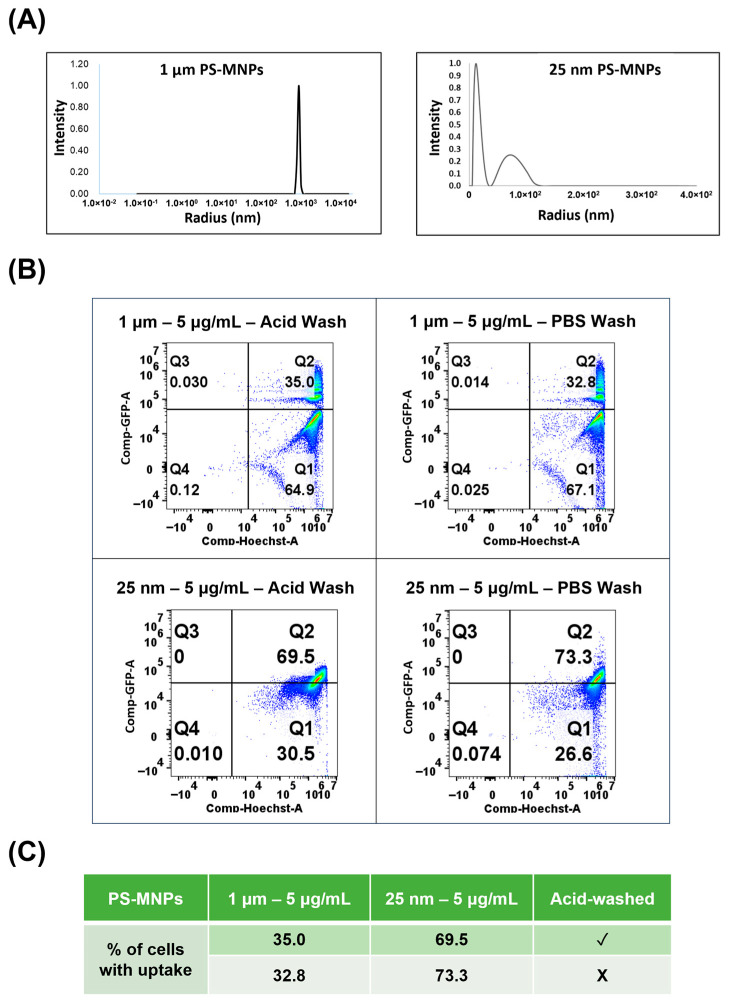
The characterization and percentage cellular uptake of PS-MNPs. (**A**) 1 µm and 25 nm sizes of PS-MNPs were measured using dynamic light scattering (DLS). (**B**) Quadrant plots showing expression of Hoechst and internalized MNPs. The color indicates cell density at each point, with blue and green indicating low density and orange and red indicating high density (**C**). The table shows the percentage of cells internalizing MNPs at both particle sizes, comparing detachment by trypsin–EDTA alone versus acid wash followed by trypsin–EDTA.

The size (hydrodynamic radius) and size distribution of the PS-MNPs used were characterized by dynamic light scattering (DLS). To determine the actual size and polydispersity index (PDI) of 1 µm and 25 nm PS-MNPs dispersed in astrocyte growth medium (AGM), dynamic light scattering (DLS) analysis was performed. The results indicated that the 1-micron particles were monodispersed with an average diameter of 1 micron (Figure 1A). While 25 nm-sized particles showed two peaks, one indicating monodispersed 25 nm particles (Figure 1A) and the other broad and less intense, indicating aggregation of 25 nm particles. This slight aggregation pattern could be due to increased ionic strength in the cell culture medium, and it was also observed in the published Transmission Electron Microscopy (TEM) images [34].

### 2.2. Cellular Accumulation of PS-MNPs

**Figure 2:**
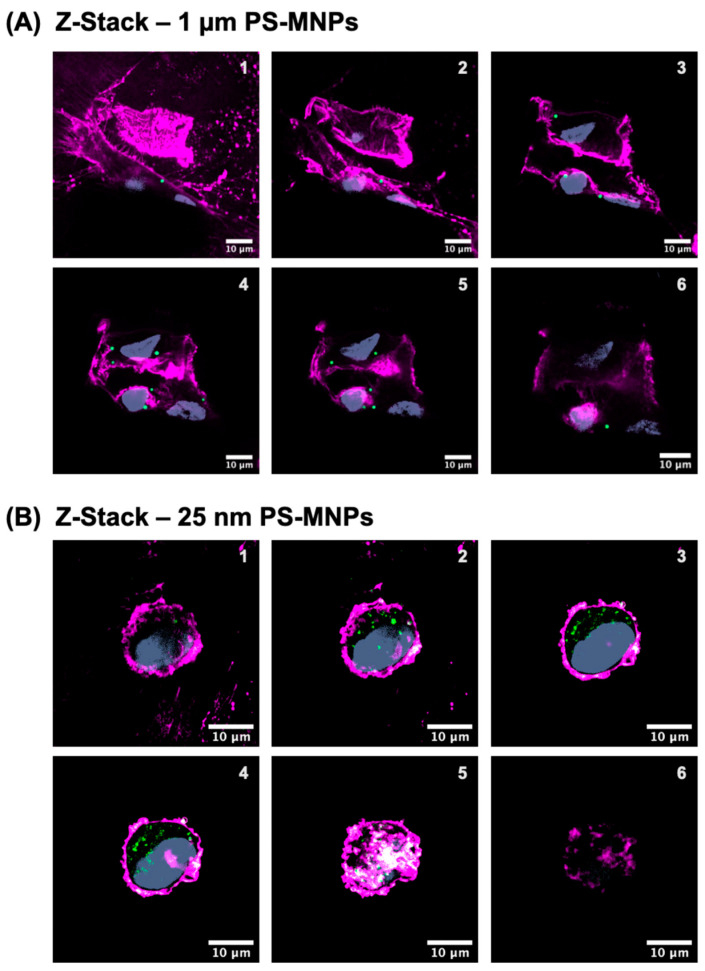
Confocal fluorescence microscopy images showing the internalization of PS-MNPs in astrocytes. (**A**) Six optical sections from the bottom to the top of cells treated with 1 µm PS-MNPs. (**B**) Six optical sections from the bottom to the top of cells treated with 25 nm PS-MNPs. Each image is 0.8 µm deep. PS-MNPs are labeled with green fluorophores. Cell membranes are stained in purple using Wheat Germ Agglutinin conjugates, and nuclei are counterstained with Hoechst 33342 (gray).

The cells were exposed to 25 nm and 1 μm PS-MNPs at 5 μg/mL for 48 h (Figure 1B,C and Figure 2 and Supplementary Figure S1). After 48 h, the internalized PS-MNPs were quantified using flow cytometry. The uptake of 25 nm particles was higher than that of 1 µm, suggesting a size-dependent uptake of PS-MNPs. Around 33% of the population was able to internalize 1 μm-sized particles, while around 70% of the population was able to uptake 25 nm MNPs. Confocal microscopy images further revealed greater accumulation of the nanosized particles within individual cells. (Figure 2). This data indicated that astrocytes were able to uptake both sizes of PS-MNPs. Therefore, the effects of these internalized particles were investigated further.

### 2.3. Cytotoxic Effects of the Exposed PS-MNPs on Cellular Viability and LDH Release

**Figure 3:**
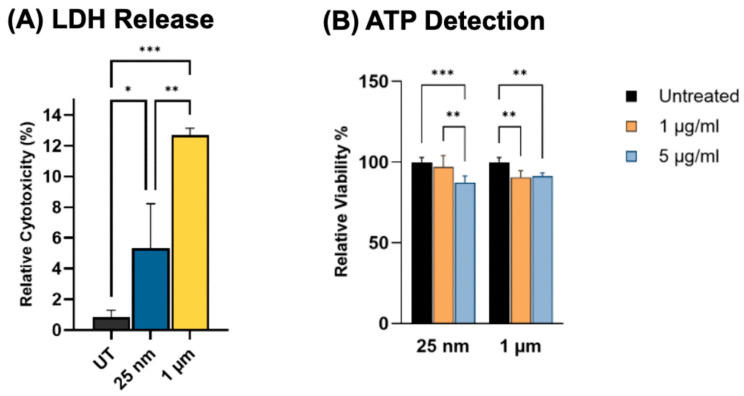
PS-MNPs induce cytotoxicity and reduce astrocyte viability. (**A**) Relative cytotoxicity was assessed by lactate dehydrogenase (LDH) after exposure to both particle sizes at 1 µg/mL. (**B**) Relative cell viability measured by ATP detection assay after 48 h of exposure to 25 nm or 1 µm PS-MNPs at 1 µg/mL and 5 µg/mL. Data are presented as mean ± SD; statistical significance is indicated as * *p* < 0.05, ** *p* < 0.01, and *** *p* < 0.001.

To evaluate whether internalized PS-MNPs cause cellular damage, the cellular cytotoxicity was investigated using the Lactate Dehydrogenase (LDH) assay. The data (Figure 3A) indicated significantly higher LDH release by astrocytes upon exposure to 1-µm MNPs (*p* < 0.01) than 25 nm MNPs. This suggests that larger MPs impose greater physical stress on astrocyte membranes, whereas smaller nanoparticles, which are more readily internalized, cause less pronounced cytotoxicity. At 48 h, PS-MNP exposure modestly reduced cell viability relative to untreated, as shown in Figure 3B. The 25 nm particles at 5 µg/mL produced a statistically significant decline (*p* < 0.01) compared to untreated and 1 µg/mL. The 1 µm particles, at both doses, caused a significant decrease in viability compared to untreated (*p* < 0.01).

### 2.4. Differential Induction of Antioxidant Defense Genes by PS-MNPs

**Figure 4:**
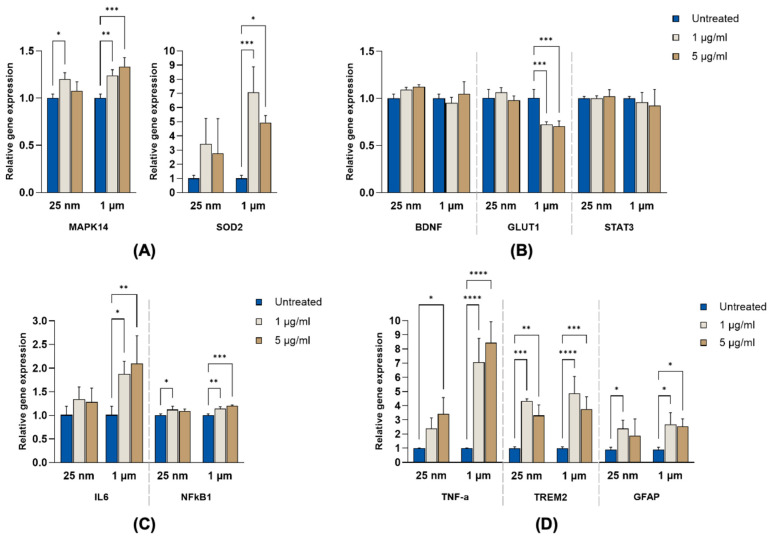
PS-MNPs differentially modulate stress-, metabolic-, and inflammation-related genes in primary astrocytes. Cells were exposed to PS particles (25 nm nanoparticles or 1 µm microparticles) at concentrations of 1 µg/mL or 5 µg/mL for 48 h; untreated cultures served as controls. Relative mRNA levels were quantified by RT-qPCR, normalized to ACTB (Beta-actin), and are plotted as mean ± SD (*n* = 3). (**A**) Oxidative–stress markers MAPK14 (p38α) and SOD2; (**B**) BDNF, GLUT1, STAT3 markers; (**C**) cytokine/transcription–factor pair IL-6 and NF-κB1; (**D**) innate-immune and reactivity markers, TNF-α, TREM2, and GFAP. A two-way ANOVA followed by a Tukey post hoc test was performed to determine the significance (* *p* < 0.05, ** *p* < 0.01, *** *p* < 0.001, **** *p* < 0.0001).

The RT-qPCR (Reverse Transcription Quantitative Polymerase Chain Reaction) results revealed significant upregulation of both MAPK14 and SOD2 in most conditions in which they were exposed to polystyrene particles. For MAPK14, both 25 nm (1 µg/mL) and 1 µm particle exposures led to a significant increase in expression compared to untreated controls (*p* < 0.05 and *p* < 0.01, respectively) (Figure 4A). For SOD2, expression was markedly elevated in response to 1 µm particles, with the high fold change observed in its low dose group (*p* < 0.001 vs. control). The 25 nm particles induced a milder increase in SOD2 expression, which did not reach statistical significance. These results indicate that MNPs exhibit size-dependent effects on oxidative stress-related genes.

### 2.5. Suppression of GLUT1 Gene Expression

BDNF expression remained largely unchanged across all treatment conditions compared to untreated controls, suggesting that neurotrophic signaling was not significantly affected by PS-MNP exposure. In contrast, GLUT1 expression was significantly reduced in response to 1 µm particle exposure. Both 1 µg/mL and 5 µg/mL treatments led to a marked downregulation of GLUT1 (*p* < 0.01 and *p* < 0.001, respectively), indicating potential disruption of glucose transport in astrocytes (Figure 4B). STAT3 mRNA levels remained unchanged under all treatment conditions. However, this finding does not exclude the possibility of STAT3 activation at the protein level. A more conclusive assessment of STAT3 involvement in the astrocytic response to PS-MNPs will require future analyses of total and phosphorylated STAT3 expression, nuclear localization, and downstream target activation.

### 2.6. Upregulation of Genes Associated with Pro-Inflammation and Reactive Phenotype

Relative mRNA expression of IL-6, NF-κB1, TNF-α, TREM2, and GFAP was assessed in astrocytes following exposure to PS-MNPs at both concentrations. IL-6 (Figure 4 and Supplementary Figure S2) expression showed a marked and statistically significant upregulation in response to 1 µm particles, with both 1 µg/mL and 5 µg/mL treatments inducing elevated expression levels compared to untreated controls (*p* < 0.05 and *p* < 0.01, respectively) as visualized in Figure 4C. No significant changes were observed in IL-6 expression following 25 nm particle exposure, although a mild upward trend was noted. NF-κB1 expression was moderately but significantly upregulated, with a slight increase noted with 25 nm 1 µg/mL (*p* < 0.05) and in both 1 µm treatment groups compared to the control (*p* < 0.01 and *p* < 0.001) (Figure 4C). TNF-α expression exhibited a robust upregulation in response to 1 µm particles. Both 1 µg/mL and 5 µg/mL treatments led to a highly significant increase compared to untreated controls (*p* < 0.0001) (Figure 4D). TREM2 expression was also significantly elevated across both particle sizes, with the strongest upregulation observed in the 1 µm low-dose group (*p* < 0.0001). GFAP, a marker of astrocyte reactivity, was significantly increased for both particle sizes (*p* < 0.05), suggesting induction of a reactive phenotype (Figure 4D). These results indicate a pro-inflammatory transcriptional response of astrocytes to PS-MNPs, particularly with the larger size, implicating the activation of astrocytes with elevated stress response and cytokine release (Figure 4 and Supplementary Figure S2).

### 2.7. Protein Level Expression Patterns of the Significant Genes

**Figure 5:**
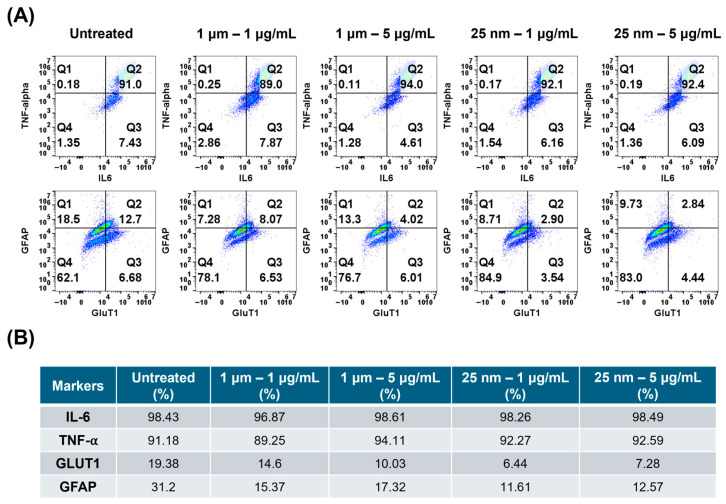
Flow cytometry analysis of astrocyte responses to PS-MNP exposure. (**A**) Representative flow cytometry quadrant plots showing intracellular cytokine and protein expression in astrocytes treated with 1 µm or 25 nm PS-MNPs at 1 µg/mL and 5 µg/mL for 48 h. IL-6 and TNF-α double staining (top row) and GLUT1 and GFAP double staining (bottom row) were analyzed to evaluate inflammatory and metabolic responses. The color indicates cell density at each point, with blue and green indicating low density and orange and red indicating high density. (**B**) The table indicates the percentage of cells positive for each marker from (**A**).

The markers that showed significant changes at the mRNA level, including inflammatory markers such as IL-6, TNF-α, and GFAP, as well as markers involved in metabolism, particularly glucose transport, such as GLUT1, were further investigated at the protein level using flow cytometry (Figure 5). Above 90% of cells expressed IL-6 and TNF-α at the intracellular level, indicating the inflammatory state of these cells (Figure 5). Upon MNPs exposure, the number of cells expressing these cytokines did not change significantly. Conversely, the number of cells expressing GFAP significantly decreased upon exposure to both sizes of MNPs, indicating the dysregulation of the post-transcriptional mechanisms. Significant changes in the expression of GFAP at both mRNA and protein levels suggest disruption of both phenotypic and functional characteristics of astrocytes. Similarly, GlUT1, the protein involved in transporting glucose across the cell membrane and the blood–brain barrier, was highly downregulated at both mRNA and protein levels. The number of cells expressing GLUT1 decreased significantly upon exposure to MNPs (Figure 5), suggesting the effect of PS-MNPs on glucose transport. Due to its crucial role in glucose transport, GLUT1 deficiency is associated with a rare genetic disorder known as GLUT1 deficiency syndrome [35], suggesting a critical role of this transporter. Overall, the protein data indicate a major change in the expression of GLUT1 and GFAP markers upon MNPs exposure.

## 3. Discussion

Increasing evidence links micro- and nanoplastic (MNP) pollution to oxidative, metabolic, and immune dysfunctions in various species. However, there is a limited understanding of their cytotoxicity towards human brain cells. To address this gap, we investigated the effects of primary human astrocytes after exposure to polystyrene particles of two clinically relevant sizes (25 nm and 1 µm) at doses of 1 µg/mL and 5 µg/mL [36]. These two sizes represent distinct microplastic and nanoplastic categories, but intermediate sizes (e.g., 100–900 nm) may exhibit nonlinear or “threshold” effects due to differences in surface area and cellular uptake. Future work will include a broader size range to determine whether such thresholds exist.

Particle uptake was first confirmed and quantified using fluorescent PS-MNPs through flow cytometry and confocal microscopy (Figure 1 and Figure 2). Glycine-HCl acid wash [37] and Trypsin-EDTA dissociation are critical for removing nonspecifically bound particles that adhere to the cell via non-covalent interactions or are trapped in surface proteins. Notably, cells exposed to 25 nm particles contained a greater number of intracellular particles, which frequently formed aggregates within the cytoplasm. Previous studies have shown that 25 nm PS nanoparticles can enter cells via both passive membrane penetration and active endocytic pathways, subsequently localizing within lysosomes [38]. In contrast, 1 µm particles are too large for classical clathrin- or caveolin-mediated endocytosis, which typically internalize cargos smaller than 200 nm [39]. Instead, their uptake is mainly mediated by actin-dependent macropinocytosis or phagocytosis-like processes, which can engulf micron-sized particles into large endocytic vesicles [40,41].

The ATP assay revealed a modest reduction in overall cell viability after 48 h of PS-MNP exposure for both particle sizes (Figure 3). While the lower dose did not produce a significant effect, the higher dose caused a notable decrease in viability in the 25 nm treatment group. Consistently, LDH assays demonstrated cytotoxic effects for both particle sizes, with 1 µm PS-MNPs inducing significantly higher LDH release than the 25 nm particles (Figure 3). Some studies demonstrate that microplastic beads in the 1–10 μm range can directly attach to and stretch lipid bilayer membranes, leading to mechanical disruption [42]. This physical membrane stress may result in membrane injury when the particles are too large to be fully internalized. Incomplete phagocytosis or persistent particle binding at the cell surface can cause leakage of lysosomal or oxidative contents, thereby damaging the plasma membrane, as reported in previous studies [43,44]. Therefore, this effect may be attributed to pronounced membrane deformation and mechanical stress caused by larger particles during the uptake process [30]. These results align with previous reports of plastic-particle toxicity in neural tissues, including mouse mixed neuronal cultures and rat NSC (neural stem cell)-derived astrocytes, as well as in other cell types such as murine fibroblasts, human lung epithelial cells, and intestinal models [18,27,29,45,46,47].

Once internalized, MNP exposure can cause activation of cellular stress pathways (e.g., oxidative stress and DNA damage), leading to cell-cycle arrest [16,48]. In our study (Figure 4), exposure to 1 µm PS-MPs at both 1 µg/mL and 5 µg/mL for 48 h significantly upregulated MAPK14 and SOD2 expression compared with untreated controls. This indicates that astrocytes mount an immediate stress response to MNPs exposure. MAPK14, a central component of the p38 MAPK pathway, is typically activated by environmental stressors, while SOD2, a mitochondrial antioxidant enzyme, is induced to counteract reactive oxygen species (ROS) generated under such conditions [49,50]. An enriched MAPK signaling pathway was also observed in a rat NSC-derived astrocyte model exposed to PS-MPs [27]. The significant concurrent increase in both markers indicates that PS-MPs induce oxidative stress in astrocytes [51]. Although 25 nm PS particles also induced mild changes in these markers, the effects were less pronounced, aligning with prior observations that larger particles elicit stronger oxidative stress [27].

In addition to oxidative stress, PS-MNP exposure triggered inflammatory responses in astrocytes. The 1 µm PS microplastics significantly upregulate pro-inflammatory genes (IL-6, TNF-α) and more moderately upregulate the transcription factor NF-κB, consistent with recent transcriptomic studies [27]. Similar results were observed in mouse models, where 1 µm PS particles activated NF-κB-mediated cytokine expression [10]. Transcriptomic profiling of rat NSC-derived astrocytes identified IL-6 as one of the most up-regulated cytokine genes after PS-MP exposure [27]. Mechanistically, TNF-α can enhance IL-6 transcription through NF-κB1-dependent pathways, forming a feed-forward loop that sustains astrocyte activation and amplifies inflammatory signaling [52,53]. The coordinated upregulation of IL-6, TNF-α, and NF-κB thus reflects a tightly coupled pro-inflammatory cascade, a pattern consistently reported in both micro- and nanoplastic exposure studies [20,54,55].

Notably, TREM2, a receptor known to promote anti-inflammatory responses in glial cells, was also upregulated. Since TREM2 typically exerts inhibitory effects on astrocytic activation, this increase may represent a compensatory mechanism aimed at counterbalancing the heightened pro-inflammatory state [56,57,58,59]. The significant elevation of GFAP expression observed across both particle sizes further supports astrocyte reactivity to PS particle exposure. This response aligns with previous reports showing that prolonged PS particle exposure promotes astroglial activation, as indicated by increased GFAP expression [30]. However, at the protein level, very few cells expressed GFAP after exposure to MNPs, possibly due to acute astrocyte damage [60]. Collectively, these results suggest that PS-MNP exposure not only provokes inflammatory signaling but also elicits feedback mechanisms to mitigate sustained activation.

In parallel, we observed a significant reduction in GLUT1 expression following exposure to these particles at both concentrations. This downregulation is consistent with the earlier findings that microplastics exhibit distinct cellular transport behaviors, leading to greater metabolic perturbation [27,61,62]. A similar repression of GLUT1 after polystyrene exposure has been reported in non-mammalian models [63]. Moreover, transcriptomic analysis in rat astrocytes revealed the downregulation of pathways involved in lipid metabolism and fatty acid oxidation, processes that can indirectly alter glucose transporter dynamics, such as those mediated by GLUT1 [27]. Supporting this metabolic link, mice fed a high-fat diet and exposed to PS-MPs (0.5–100 µm) showed reduced AKT1 expression, a key regulator of GLUT1 gene expression, compared with high-fat diet controls [64,65]. Together, these findings indicate that PS-MNP exposure impairs astrocytic glucose metabolism, potentially through alterations in signaling pathways that regulate glucose transporter expression.

In summary, these findings reveal that PS-MNPs disrupt astrocytic function through intertwined oxidative, inflammatory, and metabolic pathways, potentially undermining their role in neural homeostasis. Although the in vitro system cannot capture the complexity of the brain, this study provides mechanistic insight into astrocytic vulnerability to MNPs and underscores the need for in vivo validation to assess their contribution to neuroinflammation and glial-mediated neurodegeneration.

## 4. Materials and Methods

### 4.1. Culturing Human Astrocytes

The human astrocyte (HA) cell line (Cell Applications Inc., San Diego, CA, USA Item # 882AK-05f) was cultured in AGM (Astrocyte Growth Medium) (Cell Applications Inc., cat# 821-500). Upon reaching confluency, a portion of the cells was plated for experiments, while the remainder was cryopreserved in a freezing medium containing 90% Fetal bovine serum (FBS) and 10% Dimethyl sulfoxide (DMSO). Surface-treated culture dishes were used for all passage and treatment steps to ensure optimal astrocyte attachment. All cultures were maintained at 37 °C in a humidified incubator with 5% CO_2_.

### 4.2. Polystyrene Particles

The study utilized plain polystyrene micro- and nanoparticles (PS-MNPs) of sizes 1 µm (Phosphorex, Hopkinton, MA, USA Lot # 30210; Cat. # 112) and 25 nm (Phosphorex, Lot # 107164; Cat. # 101), as well as green, fluorescent PS-MNPs of the same sizes (Abvigen Inc., Skillman, NJ, USA 1 µm Cat. # ABWG-21-0100; 25 nm Cat. # ABWG-21-00025). Stock solutions were prepared at 1 mg/mL and sonicated for 30 min using a Bransonic^®^ M1800 ultrasonic cleaner (Emerson, St. Louis, MO, USA). Working suspensions (1 µg/mL and 5 µg/mL) were then prepared in astrocyte growth medium and vortexed for 10 s immediately prior to application to the cells.

### 4.3. Dynamic Light Scattering (DLS) Analysis of PS-MNPs

To determine the actual size and polydispersity index (PDI) of 1 µm and 25 nm PS-MNPs dispersed in AGM, dynamic light scattering (DLS) analysis was performed. Measurements of the hydrodynamic radius were calculated using the Stokes–Einstein equation implemented in the instrument software [66]. Briefly, the MNPs were first sonicated for 15 min and then were loaded into a DLS tube that had been rinsed with toluene to minimize background noise. The tube was then placed in the ALV/CGS-3 compact goniometer system (ALV-GmbH, Langen (Hessen), Germany) which was connected to the ALV-7004 correlator software (ALV-GmbH, Langen (Hessen), Germany, https://www.alvgmbh.de/Products/Software/CorrSoftware/corrsoftware.html (accessed on 18 November 2025)) to run the analysis and process the data. Prior to measurement, parameters specific to AGM were entered, including the refractive index (1.4150), angle (90°), and viscosity (0.89 cP). After the acquisition, the correlation function and distribution function were regularized. The resulting distribution graph displayed the actual hydrodynamic radius, while the polydispersity index (PDI) was obtained using the cumulant fit.

### 4.4. Nano and Microplastic Uptake by Human Astrocytes

#### 4.4.1. Quantifying Cellular Uptake Percentage of PS-MNPs

The astrocytes were cultured in Astrocyte growth medium (AGM) containing green, fluorescent PS-MNPs (5 µg/mL) of both 1 µm and 25 nm sizes, with untreated cells serving as controls. After 48 h, the cells were washed twice with a cold 0.2 M glycine buffer (pH 3.0, containing 0.15 M NaCl) for 30 s each, followed by a cold PBS wash for 30 s to ensure removal of membrane-bound particles. The cells were then detached using trypsin-EDTA to further eliminate any remaining bound MNPs. Parallel control samples were prepared in the same way, except that the acidic buffer wash step was omitted. The detached cells were washed twice with PBS to remove any uninternalized fluorescent particles. They were stained with Hoechst for 10 min and washed three times with PBS to remove excess dye and uninternalized fluorescent particles. An unstained control sample was also prepared for flow cytometry gating. All samples were analyzed using a Cytek Aurora Spectral Cytometer (Cytek Biosciences, Inc., Fremont, CA, USA), and the data were processed with FlowJo 10.8.1 software. A small portion of the treated cells was fixed and imaged using a Keyence BZ-X810 fluorescence microscope (Keyence Corporation of America, Itasca, IL, USA). A three-dimensional projection of the 25 nm nanoparticle treatment was reconstructed from Z-stack images in ImageJ (win64), as presented in Supplementary Video S1.

#### 4.4.2. Confocal Microscopy

Cells were seeded onto glass coverslips in six-well plates and incubated for 16 h. Polystyrene plastic particles (5 µg/mL) were then added and cultured for 48 h. After treatment, cells were washed sequentially with phosphate-buffered saline (PBS) and acidic buffer (0.2 M glycine–0.15 M NaCl, pH 3.0) in the order PBS–PBS–acid–PBS–acid–PBS to remove surface-bound particles. Each acid wash was limited to 30 s to prevent cell detachment. Cells were then fixed with 4% formaldehyde for 15 min at room temperature and stained with Wheat Germ Agglutinin–Tetramethylrhodamine (Thermo Fisher Scientific, Waltham, MA, USA, Cat. # W849) and Hoechst 33342 (Thermo Fisher Scientific, Cat. # H1399) lab was sequentially washed with phosphate-buffered saline (PBS) and sequentially washed with phosphate-buffered saline (PBS) and an els the plasma membrane and nuclei, respectively. Coverslips were mounted using Fluoromount-G™ (Invitrogen, Cat. # 50-187-88), and fluorescence images were captured with a Nikon CSU-W1 spinning-disk confocal microscope (Nikon Instruments Inc., Melville, NY, USA).

### 4.5. Measuring Cellular Cytotoxicity

Lactate dehydrogenase (LDH) release was measured as an indicator of cellular cytotoxicity and cytolysis using the CyQUANT™ LDH Cytotoxicity Assay Kit by Invitrogen (Cat. No. C20301). LDH catalyzes the conversion of lactate to pyruvate, accompanied by the reduction of NAD^+^ to NADH. This reduction can be utilized to reduce tetrazolium salt, generating a colored formazan product that can be quantified by absorbance. We seeded 10,000 cells per well in triplicate in 96-well plates and incubated overnight in astrocyte medium. Cells were then exposed to 1 µg/mL PS particles (25 nm or 1 µm) for 48 h. Control conditions included: untreated cells in complete medium; wells containing medium only (background); wells with serum-free medium; and wells designated for spontaneous and maximum LDH release. One hour before assay completion, 10 μL of 10× lysis buffer was added to the maximum-release wells; all other wells received 10 μL of nanopure water. After 1 h, 50 µL of supernatant from each well (including spontaneous, maximum, and serum-free controls) was transferred in triplicate to a 96-well assay plate. We added 50 μL of assay reagent, mixed gently, and incubated at room temperature for 10 min, protected from light. The reaction was stopped with 50 μL of stop solution, mixed gently, and the absorbance was measured at both 490 nm and 680 nm using a SpectraMax iD5 Multi-Mode Microplate Reader (Molecular Devices, San Jose, CA, USA). The LDH activity in each well was first calculated by subtracting the 680 nm absorbance (plate and instrument background) value from the 490 nm absorbance. Media background and serum-only signals were subtracted from spontaneous, maximum, and treated sample readings before calculating the relative cytotoxicity percentage using Equation (1).

(1) $$\mathrm{Relative}\;\mathrm{cytotoxicity}\;\mathrm{percentage}\;\left(\%\right)=\frac{\mathrm{OD}\;\mathrm{Sample}-\mathrm{OD}\;\mathrm{Spontaneous}}{\mathrm{OD}\;\mathrm{Maximum}-\mathrm{OD}\;\mathrm{Spontaneous}}\;\times \;100\;$$

### 4.6. Luminescent Assay for Cell Viability

Cells were seeded into a standard 96-well plate in both treated and untreated (control) conditions, with five biological replicates (quintuplicates) per condition. After incubation for 48 h under standard culture conditions, cell viability was assessed using the CellTiter-Glo^®^ 2.0 Cell Viability Assay (Promega, Singapore) following the manufacturer’s instructions. Briefly, after 48 h incubation, the plate was allowed to equilibrate to room temperature (30 min), then an equal volume of CellTiter-Glo reagent was added to each well. The plate was gently shaken for 2 min to promote cell lysis, then incubated at room temperature for 10 min to stabilize the luminescent signal. Subsequently, the lysates were transferred to an opaque (white) 96-well plate to minimize signal crosstalk, and luminescence was measured using a SpectraMax iD5 Multi-Mode Microplate Reader (Molecular Devices, San Jose, CA, USA). Signal integration times and read height were set according to the optimized instrument settings for luminescence mode. Percent relative viability was calculated from the relative luminescence (RLU) measured in each condition using the Formula below.

(2) $$\mathrm{Relative}\;\mathrm{viability}\;\mathrm{percentage}\;\left(\%\right)=\frac{\mathrm{RLU}\;\mathrm{Treated}-\mathrm{RLU}\;\mathrm{Background}}{\mathrm{RLU}\;\mathrm{Untreated}-\mathrm{RLU}\;\mathrm{Background}}\;\times \;100\;$$

### 4.7. Reverse Transcription Quantitative Polymerase Chain Reaction (RT-qPCR)

The total mRNA was isolated from each condition using the E.Z.N.A.^®^ Total RNA Kit I (Omega Bio-tek, Norcross, GA, USA), then concentrated and purified with the RNA Clean & Concentrator-5 Kit (Zymo Research, Irvine, CA, USA). For reverse transcription, approximately 1 µg of total RNA was anchored on oligo-dT primers and converted into complementary DNA (cDNA) using Superscript™ III Reverse Transcriptase (Invitrogen, Carlsbad, CA, USA). Primers specific to the target cDNAs (Supplementary Table S3) were designed using the Primer-BLAST tool [67], and their melting temperatures were verified using NetPrimer (http://www.premierbiosoft.com/netprimer/ (accessed on 9 July 2025)). ACTB (Actin Beta) was used as the endogenous control. qPCR was conducted using an ABI 7500 instrument (Applied Biosystems, Foster City, CA, USA) with SYBR Green PCR Master Mix (QuantaBio, Beverly, MA, USA). The thermal cycling conditions were as follows: 95 °C for 10 min, followed by 40 cycles of 95 °C for 15 s, 55 °C for 30 s, and 68 °C for 30 s. Cycle threshold (Ct) values of target genes were normalized to the Ct values of ACTB to yield ΔCt. Relative gene expression was quantified as fold change in the genes of interest, calculated using Formula (3).

Fold change = 2^−(ΔCt^_sample_^− ΔCt^_control_^)^ (3)

### 4.8. Quantitative Analysis of Marker Expressions Using Flow Cytometry

To prepare flow cytometry samples, the cells were detached with Accutase and then fixed in 10% neutral buffered formalin (VWR) for 30 min at room temperature. After fixation, the cells targeted intracellular markers were permeabilized using 100% cold methanol for 10 min. Once permeabilized, the cells were washed twice with PBS and treated for 40 min with a blocking buffer containing 2% FBS in PBS to obstruct non-specific binding sites. All samples were incubated overnight at 4 °C with the primary antibodies of interest. The following day, the primary antibodies (Supplementary Table S2A) were removed, and the cells underwent 2–3 washes with PBS. Secondary antibodies (Supplementary Table S2B) were added, and the samples were incubated for 40 min at room temperature. After another set of 2–3 washes with PBS, the samples were analyzed using a Cytek Aurora Spectral Cytometer. The results were compared against isotype controls using FlowJo software.

### 4.9. Statistical Analysis

Each experiment was performed in triplicate or quintuplicate, and results are presented as mean ± standard deviation. Statistical analysis was performed using one-way and two-way analysis of variance (ANOVA) with particle size (25 nm vs. 1 µm) and concentration (0, 1, 5 µg/mL) as fixed factors in GraphPad Prism 10. Where ANOVA indicated significance (*p* < 0.05), Tukey’s multiple comparisons post hoc test was used to compare each treatment group with the control. Graphs were generated, and statistical results were reported using Prism.

## 5. Conclusions

This study demonstrates that polystyrene micro- and nanoplastics (PS-MNPs) induce cytotoxic effects in primary human astrocytes by disrupting oxidative, inflammatory, and metabolic pathways. Both 25 nm and 1 µm PS particles were efficiently internalized, yet their biological impacts differed by size. Exposure to 1 µm PS-MPs triggered stronger oxidative stress responses, evidenced by the upregulation of MAPK14 and SOD2, and promoted a robust pro-inflammatory cascade involving IL-6 and TNF-α. Concurrently, GLUT1 downregulation and decreased GLUT1-positive cell populations indicate impaired glucose metabolism, while the increase in GFAP transcripts alongside reduced GFAP-positive cells suggests cytoskeletal dysregulation at the protein level. Collectively, these findings reveal that PS-MNPs elicit metabolic and structural dysfunctions, highlighting a shift toward a reactive but compromised phenotype. Future in vivo investigations are needed to elucidate how these astrocytic alterations by microplastic exposure contribute to neuroinflammation and the broader neuropathological consequences.

## Data Availability

The original contributions presented in this study are included in the article/Supplementary Material. Further inquiries can be directed to the corresponding author.

## Supplementary Materials

The following supporting information can be downloaded at: https://www.mdpi.com/article/10.3390/ijms262311273/s1.
