# Supplementary material for: Polystyrene Micro- and Nanoplastic Exposure Triggers an Activation and Stress Response in Human Astrocytes
Source: Int J Mol Sci. 2025 Nov 21;26(23):11273. doi: 10.3390/ijms262311273 (PMC12692081; doi:10.3390/ijms262311273)
Supplement: Supplementary file 1 [file ijms-26-11273-s001.zip › ijms-3875833-supplementary.pdf]

Supplementary Information

Polystyrene Micro– and Nanoplastic Exposure Triggers an activation and Stress response in Human Astrocytes

Sonia Kiran [1,†], Uvindu Thilanka [1,†], Yu Xue [1], and Qing-Xiang Amy Sang [1,2,*]

[1]Department of Chemistry and Biochemistry, Florida State University, Tallahassee, FL 32306-4390, USA.

[2]Institute of Molecular Biophysics, Florida State University, Tallahassee, FL 32306-4380, USA.

[†] Contributed equally to this work

[*]Correspondence: qxsang@chem.fsu.edu ; Tel.: +1-850-644-8683; Fax: +1-850-644-8281

Contact information for other authors:

Sonia Kiran: skiran@fsu.edu

Uvindu Thilanka: ud24@fsu.edu

Yu Xue: yx21@fsu.edu

Supplementary Figure S1: Gating strategy for flow cytometry analysis of PS-MNP uptake by normal human astrocytes (NHA). Representative plots showing the gating approach used to distinguish NHA cells from 1 µm PS-MNPs. The first two panels illustrate the side and forward scatter plots of 1 µm PS-MNPs alone, while the subsequent panels show NHA cell populations after exposure to PS-MNPs under different treatment conditions. This gating strategy was applied to quantify cellular uptake and to exclude particle aggregates and debris, corresponding to the data presented in Figure 1.

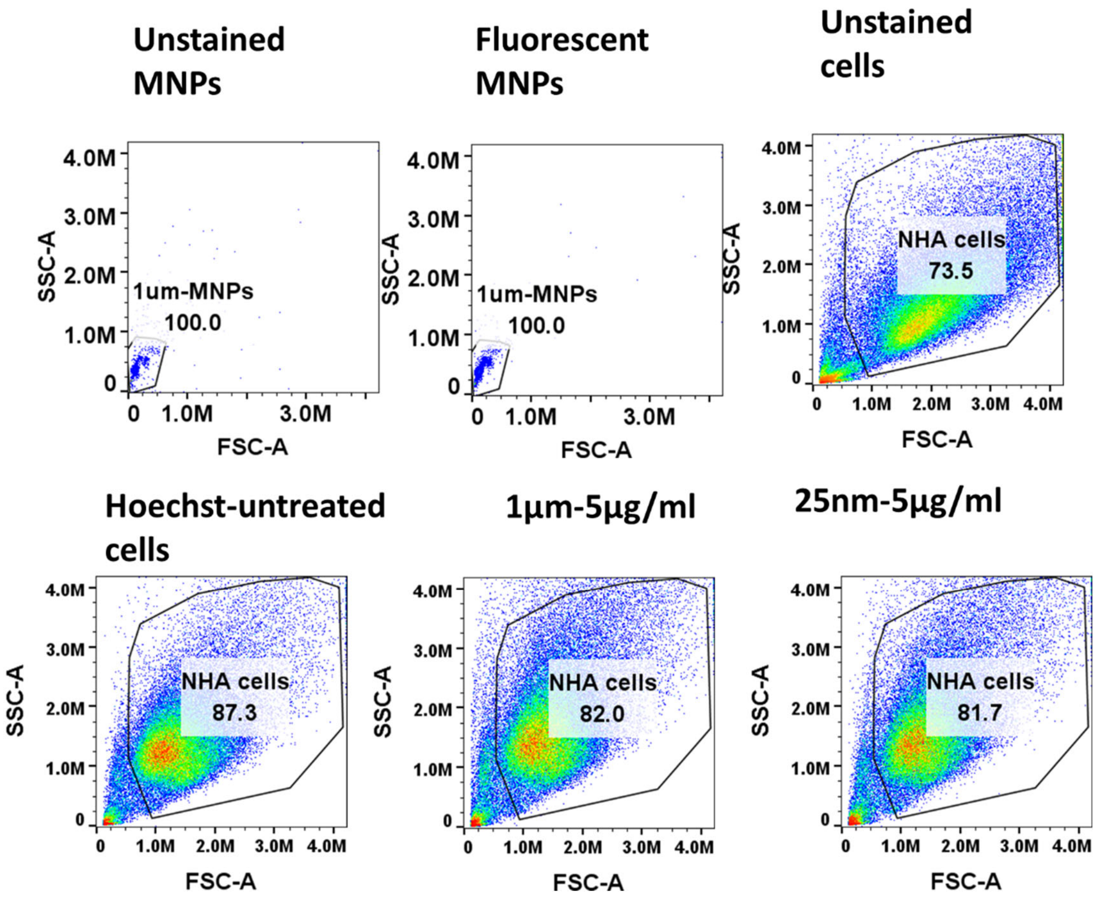

Supplementary Figure S2: Gating strategy for flow cytometry analysis of astrocyte responses to PS-MNP exposure. Representative flow cytometry plots illustrating the sequential gating used to define astrocyte populations across unstained controls and PS-MNP–treated groups (1 µm and 25 nm, 1 or 5 µg/ml). The gating strategy corresponds to the data presented in Figure 5.

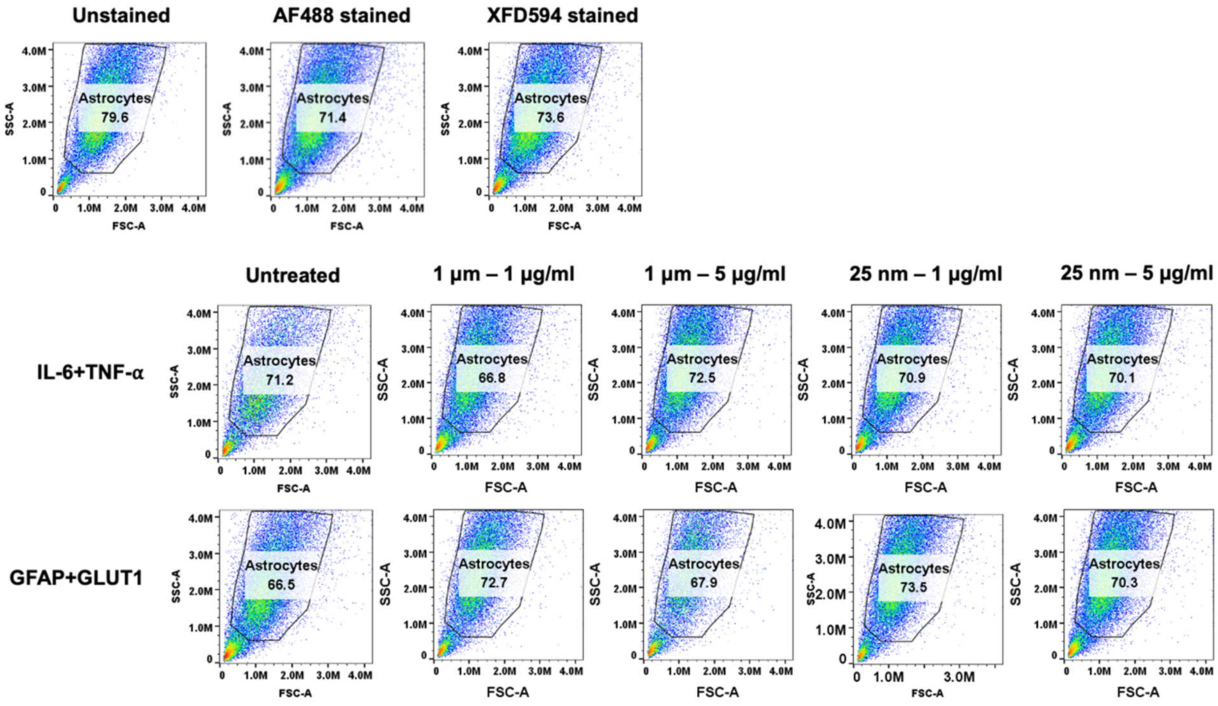

Supplementary Table S1: Polystyrene micro- and nanoplastic exposure conditions (particle size and concentration) employed in earlier neural cell in vitro experiments.

Study	Subject	Particle Size	Concentration
Hua et al., 2022 [31]	hPSC-derived cortical spheroids	1 µm, 10 µm	5, 50, 100 µg/mL
Liu et al., 2022 [34]	Mouse hippocampal neuronal HT22 cells	1 µm, 100 nm	5, 25, 75 µg/mL
Martin-Folgar et al., 2024 [28]	Human neural stem cell line (hNS1)	30 nm	0.5, 2.5, 10 µg/mL
González-Caballero et al., 2024 [32]	Human neural stem cells (NSCs)	30 nm	0.5, 2.5, 10 µg/mL
Park et al., 2024 [35]	Mouse subventricular zone NSCs	1 µm, 2 µm	10 µg/ml
Adamiak et al., 2025 [30]	Rat cerebral cortex-derived primary astrocytes & neurons	25 nm	0.5, 1, 25, 50 µg/mL

Supplementary Table S2: Antibody reagents for flow-cytometry staining. (A) Primary antibodies used for intracellular and extracellular detection of neural and inflammatory markers. **(B)** Fluorophore-conjugated secondary antibodies used to visualize mouse- and rabbit-derived primaries.

A

Target	Antibody type / Host	Supplier & Catalog #	Working dilution
GluT1	Mouse mAb		
GFAP	Rabbit pAb	Sino Biological 206278-T44	1:1000
IL-6	Mouse IgG	Sino Biological 10395-MM10	1:100
TNF-α	Rabbit IgG	Sino Biological 10602-R101	1:50

B

Fluorochrome/Specificity	Supplier & Catalog #	Working dilution
Alexa Fluor 488 Goat anti-Mouse IgG/IgM/IgA (H+L)	Invitrogen A-10667	1 : 200
XFD 594 Goat anti-Rabbit IgG (H+L)	AAT Bioquest 16404	1 : 300

Supplementary Table S3: Primer sequences used for RT-qPCR analysis of mRNA expression. F, forward primer; R, reverse primer.

Gene	Primer sequence (5'-3')
**GLUT1**, Glucose Transporter 1	F: AGCAACTGTGTGGTCCCTACG<br>R: AAGGTCCGGCCTTTAGTCTCA
**BDNF**, Brain-Derived Neurotrophic Factor	F: AATTTTGCTCCCCAGTGAAGG<br>R: GGCTCCCAACTTGACTTCTCC
**STAT3**, Signal Transducer and Activator of Transcription 3	F: CTCTCCTGTGCGTATGGGAAC<br>R: CTGAGGCAAGGTGGTTTTGAG
**MAPK14**, Mitogen-Activated Protein Kinase 14	F: AAAAGGGTCTTCTTGGCAGCTT<br>R: TCCCCATCAAAAGGAATCACA
**SOD2**, Superoxide Dismutase 2	F: CGTCATTTACAGAAGGCACACAA<br>R: TCTGAGCCCCAGTGAAGAATG
**IL-6**, Interleukin 6	F: ATGAGGAGACTTGCCTGGTGA<br>R: ATCTGCACAGCTCTGGCTTGT
**TNF-α**, Tumor Necrosis Factor-Alpha	F: CCTGGGATTCAGGAATGTGTG<br>R: TGTAGGCCCCAGTGAGTTCTG
**NFκB1**, Nuclear Factor Kappa B Subunit 1	F: TGAGTCCTGCTCCTTCCAAAA<br>R: TCGGTGTAGCCCATTTGTCTC
**TREM2**, Triggering Receptor Expressed on Myeloid cells 2	F: GTGAAGGAAGATGATGGGAGGA<br>R: TTGCCAGAGCAGAACAAGGAG
**GFAP**, Glial Fibrillary Acidic Protein	F: AGGACCTGCTCAATGTCAAGC<br>R: CAGGTTGGAGAAGGTCTGCAC

Supplementary Video S1. Three-dimensional projection of nanoparticles in primary human astrocytes. The video shows 25 nm green-fluorescent polystyrene nanoparticles in primary human astrocytes. Nuclei are counterstained with Hoechst 33342 (blue). The 3-D rendering reveals nanoparticle distribution and internalization within astrocytes. (MP4)
